# The potential role of hydrogen sulfide in cancer cell apoptosis

**DOI:** 10.1038/s41420-024-01868-w

**Published:** 2024-03-06

**Authors:** Wei Gao, Ya-Fang Liu, Yan-Xia Zhang, Yan Wang, Yu-Qing Jin, Hang Yuan, Xiao-Yi Liang, Xin-Ying Ji, Qi-Ying Jiang, Dong-Dong Wu

**Affiliations:** 1 Henan International Joint Laboratory for Nuclear Protein Regulation, School of Basic Medical Sciences, Henan University, Kaifeng, Henan 475004 China; 2 Faculty of Basic Medical Subjects, Shu-Qing Medical College of Zhengzhou, Zhengzhou, Henan 450064 China; 3 School of Stomatology, Henan University, Kaifeng, Henan 475004 China; 4 Department of Stomatology, Huaihe Hospital of Henan University, Kaifeng, Henan 475000 China

**Keywords:** Cancer metabolism, Cell death

## Abstract

For a long time, hydrogen sulfide (H_2_S) has been considered a toxic compound, but recent studies have found that H_2_S is the third gaseous signaling molecule which plays a vital role in physiological and pathological conditions. Currently, a large number of studies have shown that H_2_S mediates apoptosis through multiple signaling pathways to participate in cancer occurrence and development, for example, PI3K/Akt/mTOR and MAPK signaling pathways. Therefore, the regulation of the production and metabolism of H_2_S to mediate the apoptotic process of cancer cells may improve the effectiveness of cancer treatment. In this review, the role and mechanism of H_2_S in cancer cell apoptosis in mammals are summarized.

## Facts

- To date, H_2_S has been shown to play an important role in tumors of various tissue origins.
- H_2_S promotes cancer progression in most tissues through multiple mechanisms.
- Overexpression or knockdown of genes encoding H_2_S-producing enzymes, inhibitors of the enzymes, and H_2_S-releasing reagents have been validated in cancer cells and animal models of xenograft tumors.
- It has been reported that inhibition of H_2_S promotes apoptosis of cancer cells through a number of mechanisms.

## Open Questions

- H_2_S promotes the development of many cancer cells, including colorectal, ovarian, lung, breast and kidney. While it has opposite effects on hepatocellular carcinoma and glioma.
- What are the mechanisms by which different concentrations of H_2_S exhibit different physiological and pathological functions?
- The current detection technology of H_2_S concentration in tissues and cells is still immature.

## Introduction

**Fig. 1:**
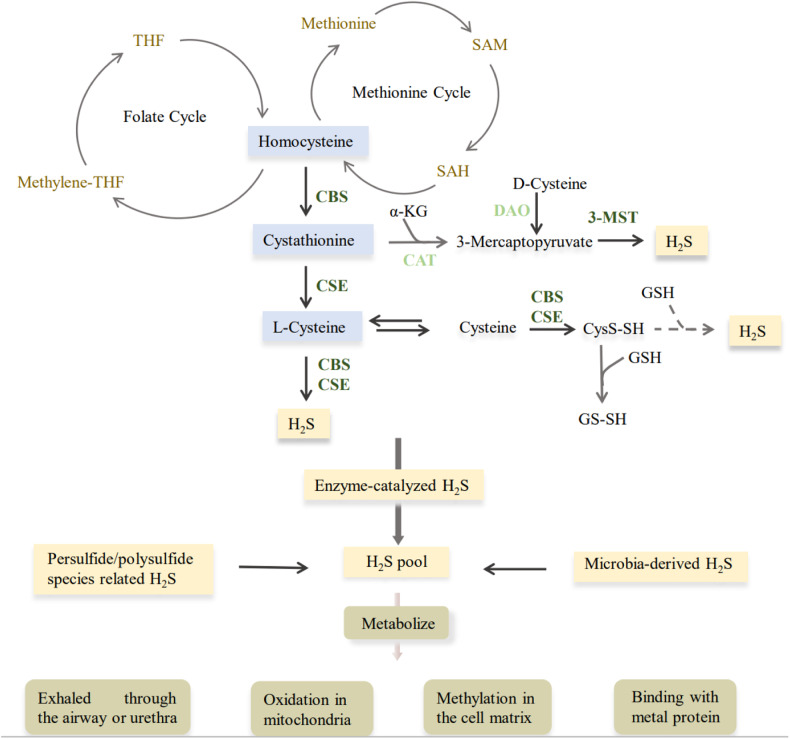
The production and metabolism of H_2_S. In mammals, H_2_S is produced endogenously from cysteine, serine, homocysteine and other substrates primarily through the actions of three major enzymes. Non-enzymatic pathways: gut microbes as well as polysulfide-derived H_2_S. Metabolism of H_2_S: H_2_S can be oxidized in the mitochondria or metabolized by methylation in the cytoplasm. H_2_S is first oxidized by SQR in the mitochondria to form a persulfide. This persulfide is further oxidized by ETHE1 to produce SO_3_^2−^, which is then converted to SO_4_^2−^ and S_2_O_3_^2−^ by sulfite oxidase and rhodan oxidase and excreted in the urine or by respiration. H_2_S can also be removed by binding to metalloproteins to form sulfheme. α-KG α-ketoglutaric acid, 3-MST 3-mercaptopyruvate sulfurtransferase, CAT cysteine aminotransferase, CBS cystathionine beta-synthase, CSE cystathionine gamma-lyase, CysS-SH cysteine persulfide, DAO D-amino acid oxidase, GSH glutathione, SAH S-adenosylhomocysteine, SAM S-adenosyl methionine, THF tetrahydrofolic acid.

**Fig. 2:**
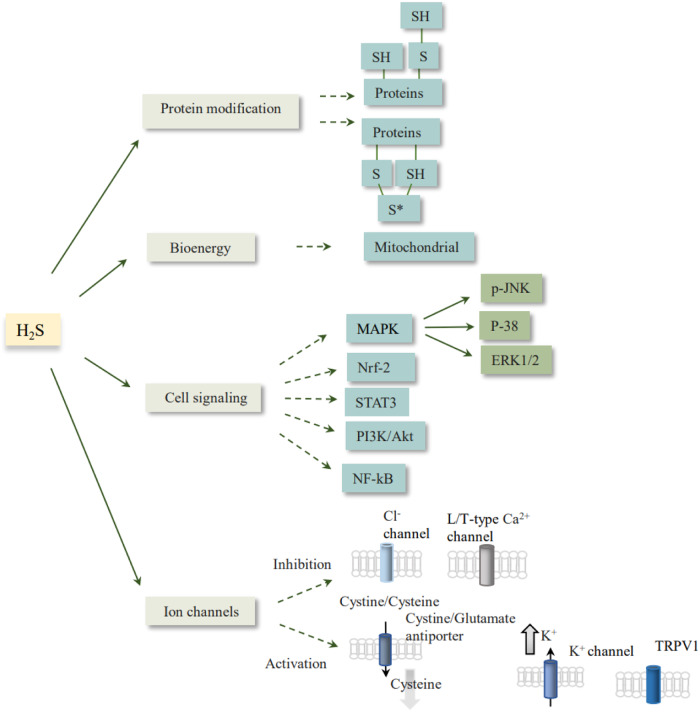
The mechanisms of H_2_S function in the organism. Promotion or inhibition of signaling pathways, post-translational modification of proteins, activation or shutdown of ion channels, and participation in mitochondrial metabolism. Akt protein kinase B, ERK extracellular signal-regulated kinase, JNK C-Jun N-terminal kinase, MAPK mitogen-activated protein kinase, NF-κB nuclear factor-kappa B, Nrf2 nuclear factor erythroid-2 related factor 2, PI3K phosphoinositide 3-kinase, STAT3 signal transducer and activator of transcription 3, TRPV1 transient receptor potential vanilloid 1.

There are currently three well-known gasotransmitters: carbon monoxide (CO), nitric oxide (NO), and hydrogen sulfide (H_2_S). Endogenous H_2_S as the third gaseous signaling molecule is produced through non-enzymatic and enzymatic desulfhydration. Non-enzymatic process is mainly through the decomposition of inorganic substances [1]. H_2_S mainly comes from different substrates catalyzed by cystathionine β-synthase (CBS), cystathionine γ-lyase (CSE), and 3-mercaptopyruvate sulfurtransferase (3-MST) [2]. The main source of H_2_S is based on S-adenosine homocysteine as a substrate, catalyzed by two pyridoxal 5’-phosphate-dependent enzymes, CBS and CSE, which are localized in the cytoplasm. 3-MST, which is localized in the cytoplasm and mitochondria, is another enzyme that catalyzes the production of H_2_S using mercaptopyruvate as a substrate. Mercaptopyruvate is produced by cysteine aminotransferase (CAT) using L-cysteine or D-amino acid oxidase (DAO) using D-cysteine [3]. In the organism, H_2_S is either metabolized directly or stored in the form of bound sulfane sulfur and acid-labile sulfur to maintain a dynamic equilibrium. Its metabolism is mainly through a series of metabolic enzymes such as mitochondrial sulfide:quinone oxidoreductase (SQR) and persulfide dioxygenase (ETHE1), which eventually produce sulfates and are excreted in the urine or through respiration [4] (Fig. 1). The metabolism of H_2_S in mammals is also involved in redox reactions, binding to heme-containing metalloproteins or post-translational modifications of proteins, and also shows the role of gasotransmitter [5–8] (Fig. 2).

**Fig. 3:**
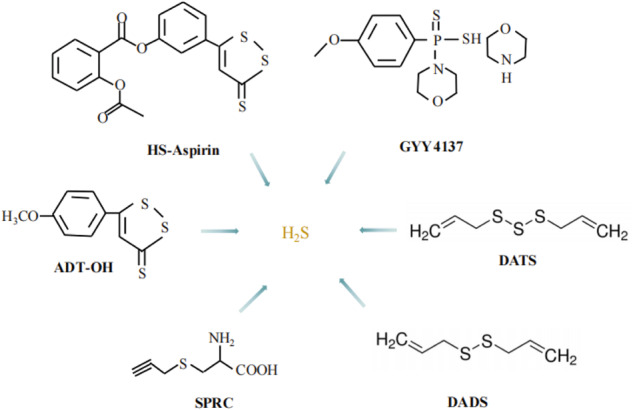
Several common H_2_S donors. DATS diallyl trisulfide, DADS diallyl disulfide, GYY4137 morpholin-4-ium 4-methoxyphenyl(morpholino) phosphinodithioate), SPRC S-propargyl-cysteine.

Three enzymes that produce H_2_S are reported to be differentially expressed in tumor tissues of different tissue origins [9] and are involved in regulating tumorigenesis and progression (Table 1). Studies have shown that its role in tumors generally exhibits a biphasic bell-shaped effect [10]. Endogenous or low concentrations of exogenous H_2_S promote cancer cell development by stimulating angiogenesis, increasing mitochondrial bioenergy, and antioxidant. While the donation of H_2_S at higher concentration prevails the suppressive bioenergetic and cytotoxic effects (Fig. 3). The H_2_S concentration above a certain threshold plays an anti-cancer role by inducing apoptosis, DNA damage, and inhibiting the cell cycle [11–18].

**Table 1:** The changes in H_2_S-producing enzymes in different types of cancer.

Cancer types	Cell lines	H_2_S producing enzymes		
		CSE	CBS	3-MST
Melanoma	A375, WM35, SK-Mel-5, Sk-Mel-28, PES43	↑	NT	↑
Colon cancer	HCT116, HT29	↑	↑	↑
Prostate cancer	LNCaP, PC3	↑	↑	NT
Gastric cancer	SGC-7901	↑	↑	NT
Ovarian cancer	OV202, SKOV3, A2780, OVCAR3, OVCAR4, OVCAR5	NC	↑	NT
Breast cancer	Hs578T, MCF7	↑	↑	NT
Renal cancer	RCC4	↑	↑	↑
Thyroid cancer	TPC1, TT, ARO	↑	↑	NC
Gliomas	C6, U87MG	NT	NT	↑
Hepatocellular carcinoma	HepG2, PLC/PRF/5	↑	↑	NT
Urothelial carcinoma	5637, EJ, UM-UC-3	↑	↑	↑
Astrocytoma	U373	NT	NT	↑
Neuroblastoma	SH-SY5Y	NT	NT	↑
Leukaemia	HL-60, MV4-11	NT	↑	NT
Biliary tract carcinoma	TFK-1, HUCCT-1, SNU308	NT	↑	NT

*NT* not tested, ↑ upregulation, *NC* no change, *CBS* cystathionine beta-synthase, *CSE* cystathionine gamma-lyase, *3-MST* 3-mercaptopyruvate sulfurtransferase.

Apoptosis is a form of programmed cell death that results in the orderly and efficient removal of damaged cells to ensure a homeostatic balance between the rate of cell formation and cell death. However, the disruption of this balancing function can contribute to an abnormal cell growth/proliferation or autoimmune disorders. Apoptosis is considered a vital component of various processes including normal cell turnover, proper development and functioning of the immune system, hormone-dependent atrophy, embryonic development and chemical-induced cell death. Deregulation in apoptotic cell death machinery is one of the hallmarks of cancer [19–21]. The concept of apoptosis is first introduced as a barrier to cancer by Kerr et al. in 1972 [22]. Tumors are new organisms formed when the cells of local tissues lose the normal regulation of growth under the action of various carcinogenic factors, leading to their clonal abnormal proliferation. This is due to the accumulation of many genetic and epigenetic changes within the cell, expressed in the accumulation of chromosomal or molecular aberrations, which leads to genetic instability. The simultaneous interaction of environmental factors, exogenous factors, and individual genetic instability factors can contribute to tumorigenesis. Among these factors, individual characteristics play the most significant role. [23, 24]. The human oncogenic gene *p53*, for example, encodes the P53 protein as a transcription factor, which is frequently mutated in human cancers and is associated with early apoptosis [25].

Escaped apoptosis is widely recognized as a prominent hallmark of cancer cells [26]. There are multiple ways in which cancer cells can reduce apoptosis or enhance apoptosis resistance. In general, the mechanisms by which cells evade apoptosis can be broadly classified as: 1) disrupted balance of pro- and anti-apoptotic proteins, 2) reduced caspase function, and 3) impaired the signal transduction of death receptor [27]. Given the importance of apoptosis in tumorigenesis and progression, targeting apoptosis-related pathways in cancer cells have emerged as promising approaches for the treatment of various tumors [28].

There are three main apoptotic pathways: intrinsic, extrinsic and endoplasmic reticulum (ER) pathways [29]. The caspase family is the core member in initiating and executing apoptosis [30]. Those factors that initiate apoptosis are: caspase 8, caspase 9 and caspase 10, the members that execute apoptosis are: caspase 3, caspase 6 and caspase 7. Caspase 8 and caspase 10 are the promoters of extrinsic apoptosis, and caspase 9 is the promoter of intrinsic apoptosis. Caspase 3 is the key enzyme of apoptosis, and its activation indicates the beginning of apoptosis execution. Other major factors involved in apoptosis are: 1. Cytochrome C (Cyt C), Cyt C binds with Apaf-1 protein in the presence of ATP to form an apoptotic complex that recruits and activates pro-caspase 9. Cyt C is also engaged in the caspase non-dependent pathway that promotes chromatin condensation and mediates apoptosis [31]. 2. Smac/Diablo and HtrA2/Omi inhibit the release of inhibitory apoptosis protein (IAP) and indirectly facilitates the release of apoptosis execution protein caspase 3/6/7. 3. Caspase activation induces the translocation of EndoG in addition to AIF into the cytosol, which leads to the subsequent characteristic features of apoptosis, including chromatin condensation and nuclear fragmentation [32–34].

I. Intrinsic apoptosis of mitochondrial pathway: DNA damage, growth factor deficiency, ROS and other endogenous stress cause the Bad/Bak proteins (respectively pro-apoptotic members of the Bcl-2 family) to form a complex, which is inserted into the mitochondrial outer membrane, changes the permeability of the mitochondrial membrane, and leads to the change of membrane potential. In addition, the Bax/Bak-mediated mitochondrial outer-membrane permeabilization is sufficient to induce the release of Cyt C, Smac/Diablo and HtrA2/Omi. While downstream caspase activation is required for the release of EndoG and AIF. Once released, Cyt C, interaction with the apoptosis protease-activating factor 1 (Apaf-1), triggers the initiator caspase 9 activation, which leads to the subsequent characteristic features of apoptosis, including chromatin condensation and nuclear fragmentation.
II. Extrinsic apoptosis by the cell surface death receptor (e.g. FasL, TARIL) pathway. After the ligand binds to the corresponding cell surface death receptor, it recruits intracellular apoptosis-associated proteins to form the death-inducing signaling complex (DISC), which contains a junction molecule and pro-caspase 8, then activates caspase 8, causing the onset of the downstream apoptotic cascade [35, 36].
III. Apoptosis of the ER pathway: The ER is the dominant site of protein synthesis and also engaged in the regulation of intracellular calcium homeostasis. When the protein is misfolded or unfolded in the cell, prolonged unfolded protein response (UPR) occurs, the transmembrane proteins PERK, IRE1 and ATF6 on the ER are responsible for protein repair. Apoptosis is triggered when the ER pressure induced by the UPR is too high [37]. ER stress activates inositol 1,4,5-trisphosphate receptor (IP3R) and/or ryanodine receptor (RyR), inducing Ca^2+^ release from ER, causing an imbalance in intracellular Ca^2+^ homeostasis, which directly induces the production of pro-apoptotic proteins of the Bcl-2 family and activates calpain, then activates csapase 9 through the activation of capase 12 [38]. In organisms, Ca^2+^ acts as a second messenger, responsible for intracellular signaling to trigger physiological changes such as apoptosis. It has also been reported that in the early stages of apoptosis, the released Cyt C binds to IP3R, causing Ca^2+^ release and inducing the calcium-dependent apoptosis [39].

There have been a number of reports on the involvement of H_2_S in the regulation of physiological apoptosis in the organism.

H_2_S affects the mitochondrial pathway of apoptosis, and at non-toxic concentrations, H_2_S accumulation in mitochondria induces mitochondria-dependent apoptosis by inducing Bax translocation, mPTP formation, and release of Cyt C [40]. H_2_S induces apoptosis through oxidative stress-triggered mitochondrial pathway in zebrafish at embryonic and larval stages [41]. Invasive bacteria in the oral cavity cause apoptosis of human pulp stem cells (HPSCs) by producing large amounts of H_2_S and activating the mitochondrial pathway [42]. However, under certain conditions, H_2_S also exhibits an inhibitory effect on apoptosis, it has been reported that H_2_S inhibits the mitochondrial K^+^_atp_/MAPK-mediated pro-apoptotic pathway [43]. In chondrocytes, NaHS-derived H_2_S may antagonize IL-3β-induced inflammation and apoptosis associated with mitochondrial dysfunction by inhibiting the PI3K/Akt/NF-κB and MAPK signaling pathways, respectively [44].

H_2_S affects TNF receptor family-mediated exogenous apoptosis: CSE-catalyzed production of endogenous H_2_S induces sulfurylation of Cys38 of the NF-κB subunit p65, which inhibits TNF-α-induced apoptosis [45].

Role of H_2_S in the apoptotic pathway of ER stress: In cervical cancer cell line Hela, morpholin-4-ium 4-methoxyphenyl(morpholino) phosphinodithioate (GYY4137), a slow-releasing H_2_S donor induces apoptosis by stimulating ER stress, causing up-regulation of IP3R1 and IP3R2 expression on the ER, leading to intracellular Ca^2+^ overload [46]. It has also been reported that H_2_S exerts a protective effect by inducing ER stress in alveolar epithelial cells in the early stages of acute lung injury in rats [47], the exact mechanism of which is unclear.

H_2_S and its producing enzymes are involved in three apoptotic pathways and play an important role in tumorigenesis and development, this could be dependent on the dose of this gaseous mediator and possibly on the differences in sensitivity of various cancer cell types to the impact of this molecule. The main objective of this review is to explore the role of H_2_S and its producing enzymes in tumorigenesis and development by mediating apoptosis.

## The roles of H_2_S in apoptosis in different tumors

GYY4137 and sodium hydrosulfide (NaHS) act as H_2_S donors and are able to release H_2_S when water-soluble. Li et al. [48] screen seven different human cancer cell lines (HeLa, HCT-116, Hep G2, HL-60, MCF-7, MV4-11 and U2OS) and normal human lung fibroblasts (IMR90, WI-38) and apply GYY4137 and NaHS, respectively, find that H_2_S donors specifically cause partial G_2_/M arrest and promote apoptosis. Another H_2_S donor sodium sulfide (Na_2_S) has also been used to selectively upregulate ROS levels in glioblastoma (GBM) cell lines (T98G and U87) to promote apoptosis and enhance their sensitivity to radiotherapy. But the effects were not observed in normal human brain microvascular endothelial cells (hCMEC/D3) [49]. NaHS induces apoptosis in C6 glioma cells by activating p38/MAPK and p53 signaling pathways in an endogenous manner [50]. In addition, the roles of H_2_S in tumor cells of different tissue origins are highlighted below.

### Lung cancer

Non-small cell lung cancer (NSCLC) A549 and 95D highly express CBS, CSE, and 3-MST compared to normal lung epithelial cell lines, induce high intracellular concentrations of H_2_S, and promote angiogenesis and epithelial-mesenchymal transition [51], which is mainly associated with the regulation of hypoxia-inducible factor-1α (HIF-1α) stimulation of vascular endothelial growth factor expression by intracellular highly concentration of H_2_S. Silencing of H_2_S-generating enzymes using siRNAs promote apoptosis, and related inhibitors of the enzymes also inhibit the growth of transplanted tumors in nude mice. CBS expression is down-regulated in A549/DDP cisplatin-resistant lung cancer cell line. After exogenous supplementation with H_2_S using NaHS (800  μM), the expression of p53 and p21, as well as apoptosis-associated proteins caspase 3 and Bax are upregulated in drug-resistant cells, which promotes apoptosis and enhances the sensitivity to cisplatin [52].

### Esophageal carcinoma

Esophageal cancer is one of the most aggressive cancers among all gastrointestinal malignancies [53]. The current main treatment for esophageal cancer is still surgical resection, but it is highly invasive and has high postoperative complications and mortality. Lei et al. demonstrate that exogenous high concentration of H_2_S (400 μM NaHS) induces cancer cell proliferation, anti-apoptosis, angiogenesis and cell migration in EC109 esophageal cells by activating the HSP90 pathway[54]. The heat shock protein HSP90 acts as a molecular chaperone that promotes the folding of ab initio synthesized or misfolded proteins, relieves the stress of UPRs in the ER, and promotes cell survival [55]. Lei et al. also find that exogenous high concentration of H_2_S (500 μM NaHS) may significantly reduces cell apoptosis by activating the JAK2/STAT3 signaling pathway, upregulating Bcl-2 and downregulating caspase 3, caspase 9, caspase 12, and Bax [56]. Current studies on the specific mechanism of action of H_2_S in esophageal cancer are scarce.

### Gastric cancer

Zhang et al. prove that [57] the expression levels of CSE and CBS proteins are significantly up-regulated in gastric cancer compared to neighboring non-cancerous tissues. Later, the same group reports exogenous administration of NaHS in human gastric cancer cell line SGC-7901 can promote Bax expression and induce apoptosis via mitochondrial pathway. In gastric cancer SGC-7901 cells, SPRC enhances the expression and enzymatic activity of CSE, which in turn acts as a substrate for CSE to cleave and produce H_2_S, and promotes apoptosis by activating the MAPK pathway to upregulate the expression of p53 and Bax [58]. Two specific inhibitors of CBS and CSE, aminooxyacetic acid (AOAA) and DL-propargylglycine (PAG), enhance the stronger anticancer effect of 3,3'-Diindolylmethane (DIM) in gastric cancer BGC-823 and SGC-7901 cells [59]. Zhu et al. use cBioPortal to analyze TCGA gastric cancer patients and find a significant association between CBS mutations and PI3K/AKT/mTOR pathway activation [60]. Further validation in gastric cancer cell lines reveals that knockdown of CBS results in excessive activation of PI3K/Akt signaling pathway and promotes oncogenic transformation.

### Hepatocellular carcinoma

Compared to normal hepatocyte line L02, human hepatocellular carcinoma cell line (e.g. PLC/PRF/5) shows significantly higher H_2_S and increased overexpression levels of CSE and CBS [61, 62], at low concentrations, 25-100 μM NaHS promotes the proliferation of hepatocellular carcinoma (HCC) cell lines, while at high concentrations, 800–1000 μM NaHS induces HCC apoptosis. High concentrations of NaHS promote the expression of PTEN, a tumor suppressor protein that inhibits PI3K/Akt signaling [63]. The overexpression of PTEN may lead to the inactivation of Akt/ERK pathway and promote apoptosis. Administration of 500 μM NaHS to PLC/PRF/5 hepatoma cells for 24 h significantly increases the expression of CSE, CBS and induces NF-κB activation, which in turn causes an increase in the expression of downstream pro-proliferative signaling molecules COX-2 and MMP-2 [62]. CSE is over-expressed in hepatoma HepG2 and PLC/PRF/5 cells, inhibition of the CSE/H_2_S axis causes elevated intracellular ROS, upregulated p53/p21 expression, and decreased Bcl-2/Bax ratio, as well as activates JNK/MAPK, which together promote the mitochondrial apoptotic pathway in HCC cells [64]. Jia et al. [65] find that knockdown of CBS in HCC cells causes an increase in intracellular ROS and induces apoptosis of cancer cells in a mitochondria-dependent manner. Zhou et al. [66] reveal that reduced CBS expression in HCC is associated with poor prognosis in HCC, and downregulation of CBS activates the IL-6/STAT3 signaling pathway, which directly inhibits apoptosis and induces infiltration of Treg cells in the tumor microenvironment. In addition, miR-24-3p is shown to be an upstream suppressor of CBS in HCC. Another endogenous H_2_S-producing enzyme, 3-MST, is down-regulated in the expression level. Overexpression of 3-MST inhibits proliferation and induces apoptosis, and 3-MST overexpression is also shown to significantly inhibit tumor growth in a nude mouse tumor allograft model, 3-MST silencing using siRNA then significantly promotes tumor growth. In addition, HCC models are more readily induced in 3-MST knockout mice, upregulation of 3-MST expression mainly causes dysregulation of intracellular ROS homeostasis and inhibits the proliferation-related AKT/FOXO3a/Rb signaling pathway [67].

### Pancreatic carcinoma

In pancreatic cancer Capan-2 cells, diallyl trisulfide (DATS), an active component of garlic that can release H_2_S, induces apoptosis by down-regulating Bcl-2, Akt and cyclin D1 protein levels, and up-regulating Bax, Fas, p53 and cyclin B protein levels [68].

### Colorectal cancer

Colorectal cancer (CRC) is the third most prevalent tumor and the second leading cause of cancer death worldwide [69]. In human intestinal lumen, there are many microorganisms involved in H_2_S production and metabolism, and H_2_S produced via microbial metabolic reactions can easily penetrate into the biofilm covering the colonic cells and epithelial cell membranes [70]. The production and metabolism of H_2_S in intestinal lumen act on CRC progression [71, 72]. It has been demonstrated that NO, CO and H_2_S, which are endogenously produced in colon cancer cells as gaseous signaling transmitters in the organism, can inhibit the proliferation of cancer cells at higher or lower concentrations, and it has been further verified that these gas signaling molecules promote apoptosis in colon cancer cells mainly by inhibiting the cGMP/VASP pathway, the Akt and ERK1/2/MAPK signaling pathways [73]. In human metastatic CRC (mCRC) cells, activation of the permeable channel transient receptor potential vanilloid 1 (TRPV1) by NaSH induces extracellular Ca^2+^ inward flow and subsequent activates the reverse mode Na^+^/Ca^2+^ (NCX) exchanger, resulting in sustained intracellular Ca^2+^ overload, which in turn induces apoptosis [74]. The expression level of cysteine-rich matricellular protein 61 (Cyr61) is higher in CRC tissues and cell lines than in normal colonic mucosa, and Cyr61 is known as an angiogenic inducer that promotes tumor growth and angiogenesis [75]. Cyr61 promotes cell migration, invasion and metastasis in CRC, and high expression of Cyr61 is associated with poor prognosis [76, 77]. Polysulfide and 3-MST-derived H_2_S promote CRC development and progression via persulfidation of Recombinant Specificity Protein 1 (Sp1) and activation of p38/MAPK to induce high expression of Cyr61, while apoptosis of cancer cells increases after application of HMPSNE to inhibit 3-MST enzyme [78]. It is known that the CBS promoter contains an Sp1 binding site, and Sp1 is essential in the control of CBS transcription [79], In colon cancer cells which devoid of p53, the chemotherapeutic drug 5-FU can induce ribosomal protein L3 (rpL3) as proapoptotic factor. RpL3 can inhibit CBS via binding with Sp1 and/or acting on post-translational. Ultimately leading to reduces H_2_S synthesis and induces Cyt C release, resulting in apoptosis of CRC cells by the mitochondrial pathway [80]. P53 is known as a tumor suppressor protein and causes tumorigenesis when it is mutated [81, 82]. P53 has a central role in the response to cellular stress, blocking the cell cycle by inducing p21 expression when cell growth is uncontrolled [83]. When damage cannot be repaired, p53 induces high expression of apoptotic proteins (e.g. BAX) to promote apoptosis [84, 85]. Caco-2 is one of the cancer cell lines that lacking p53 protein expression, and the apoptosis activation mechanism is significantly different from other CRC cell lines such as HT-29 [86, 87], apoptosis is promoted mainly by activating the extrinsic apoptotic pathway. GYY4137 induces apoptosis by blocking the cell cycle in human CRC Caco-2 cell line [88]. In other CRC cell lines (e.g. HCT116, SW620, DLD1), GYY4137 also increases the sensitivity of CRC cells to paclitaxel to promote apoptosis [89]. S-adenosyl-L-methionine (SAM), a metabolic activator at low to moderate levels of CBS, produces H_2_S as an endogenous pro-growth and bioenergetic factor in early stages of colon carcinogenesis, but high doses of SAM downregulate CBS expression and inhibit cancer cell bioenergetics and proliferation [90]. Yue et al. [91] also find that application of AOAA in colon cancer cells can inhibit endogenous H_2_S levels, disrupt cellular antioxidant capacity, increase intracellular ROS levels, upregulate p53 expression, induce apoptosis in a mitochondria-dependent manner, and enhance the sensitivity of colon cancer cells to oxaliplatin.

### Breast cancer

Inhibition of three enzymes that produce H_2_S in breast cancer (BC) cells significantly promotes apoptosis in the mitochondrial pathway [15], the mechanism is that lower intracellular levels of H_2_S inhibit phosphorylation of the PI3K/Akt/mTOR signaling pathway. The same team find that in nasopharyngeal carcinoma cells, inhibition of endogenous H_2_S induces the increase of intracellular ROS, activation of p38/MAPK and JNK/MAPK signaling pathways that contribute to apoptosis, and inhibit cell proliferation via blocking ERK1/2 MAPK [92].

### Urogenital cancer

Renal cell carcinoma (RCC) are the most common solid lesions in the kidney, accounting for approximately 90% of all renal malignancies [93]. There are three main subtypes of RCC: clear-cell RCC (ccRCC 70–80%), papillary RCC (pRCC types I and II, 10–15%) and chromophobe RCC (4–5%). Breza. Jr et al. [94] find that the expression of H_2_S-producing enzymes are downregulated in tumor tissues compared with non-tumor tissues, the levels of CBS and CSE are lower with the higher grade of ccRCC, blocking with AOAA and PAG reveals prevention of apoptosis induction.

### Female reproductive system cancer

Sanjib Bhattacharyya et al. [95] find that CBS is overexpressed in primary epithelial ovarian cancer and ovarian cancer cell lines, while knockdown of CBS disruptes intracellular energy metabolism, induces elevated ROS, promotes apoptosis and increases numbers of cancer cells killed by cisplatin via reduce NF-κB activity. Meanwhile, knockdown of CBS also significantly inhibites the growth of nude mouse tumor xenografts and enhances the inhibition of cisplatin. A team find that in ovarian cancer cell line A2780 cells, GYY4137 induces ER stress under hypoxic conditions, leading to apoptosis in a Ca^2+^-dependent manner [96]. Moreover, in ovarian cancer cell line A2780, GYY4137 induces intracellular acidification by uncoupling the highly expressed sodium-hydrogen exchanger 1 (NHE1) from sodium-calcium exchanger 1 (NCX1) [97]. It has been reported that GYY4137 upregulates NCX1 and β1, β3 adrenergic receptors in the cervical cancer cell line HeLa cells, NCX1 and β1, β3 adrenergic receptors can form a complex to enhance signaling in response to apoptosis [98].

### Hematologic neoplasms

Chronic myeloid leukemia (CML) is a malignant cancer that originates from hematopoietic stem cells [99], the critical causative event in CML is the formation of the Philadelphia chromosome, a product of a chromosomal translocation that brings together the ABL gene on chromosome 9 and the BCR gene on chromosome 22. ABL is a proto-oncogene, and after binding to the BCR gene, the downstream pathway is continuously activated, resulting in secondary abnormal leukocyte proliferation [100]. BCR-ABL induces intracellular ROS accumulation, enhances PI3K signaling, activates the transcription factor NF-κB and mediates cell transformation [101]. In CML K562 cells, CBS expression is upregulated and inhibition of CBS by shRNA or AOAA induced apoptosis in the mitochondrial pathway. In K562 cells inhibiting CBS, NF-κB activity is significantly downregulated and NF-κB failed to induce the expression of downstream target genes, causing the accumulation of ROS in cells leading to the activation of JNK and apoptosis [102].

### Melanoma

Two of the most frequently deregulated pathways in melanoma are MAPK/ERK and PI3K/Akt [103]. These two pathways play important roles in melanoma development and progression and are involved in the mechanism of resistance to the targeted therapy [104]. In A375 melanoma cells, either CSE overexpression or exogenous H_2_S donors (e.g. DATS, GYY4137) promotes apoptosis in the mitochondrial pathway, and the pro-apoptotic effect is associated with the inhibition of NF-κB downstream-related pro-survival pathways, such as reduction of the expression of c-FLIP, XIAP and Bcl-2, and inhibition of AKT and ERK1/2 signaling pathways [105]. In addition, in melanoma cells A375 and SK-MEL-28, Xiao et al. [106] find that NaHS also induces apoptosis and autophagy by inhibiting the PI3K/Akt/mTOR signaling pathway. The pleckstrin homology like domain family A member 1 (PHLDA1) is a member of three PHLDA genes. PHLDA1 can inhibit tumor development by suppressing the Akt signaling pathway [107]. H_2_S produced by bacteria in the oral cavity promotes apoptosis of cancer cells by inhibiting the expression of PHLDA1 in the tongue cancer cell line SCC-1 cells [108].

## Novel synthetic H_2_S releasing drug

In addition, for the sake of improving the pharmacological/therapeutical profiles of the clinical drugs, H_2_S-releasing moieties are introduced to the parent drugs.

### H_2_S-releasing NSAIDs

Nonsteroidal anti-inflammatory drugs (NSAIDs) are a class of medications used to treat pain, fever, and other inflammatory processes [109]. Aspirin [110] is the most commonly used NSAIDs that can inhibit cyclooxygenase (COX) activity. Cyclooxygenase-2 (COX-2) is frequently expressed in various tumors and plays a role not only in promoting tumor development but also elevating the resistance to chemotherapy and radiotherapy [111]. Aspirin exerts its anticancer effects via inhibition of COX, interference with proliferative pathways, cancer-related inflammation, and antiplatelet-driven pro-carcinogenic activity [112]. However, there is a risk of gastrointestinal and intracranial hemorrhage and other adverse effects associated with the long-term application of large amounts of aspirin. H_2_S-releasing nonsteroidal anti-inflammatory drugs (HS-NSAIDs) are an emerging class of compounds with significant anti-inflammatory properties [113]. It generally consists of a covalent linkage between traditional NSAIDs and H_2_S-releasing fractions. The HS-NSAIDs has a stronger apoptosis-inducing effect compared to traditional NSAIDs in human colon, breast, pancreatic, prostate, lung, and leukemia cancer cell lines [114]. Growing evidence indicates that HS-NSAIDs are more effective in suppressing tumors of multiple tissue types origin with less toxic side effects [115–118]. For example, the mechanism of action of NOSH-aspirin in inhibiting pancreatic cancer cells: FoxM1 promotes a transcription factor that can regulate a network of genes associated with mitosis, NOSH-aspirin inhibits FoxM1 expression via upregulation of ROS levels and p53 expression, and induction of cell cycle arrest and apoptosis [119]. HA-ADT can inhibit the proliferation, migration and invasion of human esophageal cancer cells more effectively than NaHS or GYY4137, mainly by inhibiting the PKB/Akt/mTOR pathway [120]. The thioredoxin reduction system, consisting of thioredoxin (Trx), thioredoxin reductase (TrxR), thioredoxin peroxidase (TPx), involves in cell proliferation, redox state and apoptosis, and frequently upregulate in malignancies, and can regulate the expression of transcription factor NF-κB or apoptosis signal-regulating kinase 1 (ASK-1) [121–123]. The nuclear factor NF-κB family of eukaryotic transcription factors plays an important role in regulation of immune responses, embryonic and cellular lineage development, apoptosis, cell cycle progression, inflammation and tumorigenesis [124–126]. HS-ASA induces apoptosis by inhibiting NF-κB and TrxR activity in MDA-MB-231 cells, the model of triple-negative breast cancer cells [127]. Aberrant activation of NF-κB is also present in CRC, and H_2_S-releasing naproxen induces HT-29 apoptosis by inhibiting the activation of NF-κB and TrxR [128]. ATB-346 is a novel H_2_S-NSAID that promotes apoptosis in human melanoma cells by inhibiting the activation of NF-κB and Akt [129]. ADT-OH inhibits the activation of NF-κB in B16F10 melanoma cells, reduces the expression of downstream target genes of NF-κB pathway (e.g. XIAP, Bcl-2), and induces cell apoptosis via mitochondrial pathway. In addition, ADT-OH can also elevate intracellular FADD levels and induce apoptosis in the exogenous pathway by downregulating the expression of Makorin ring finger protein 1 (MKRN1), the E3 ubiquitin ligase of FADD [130]. Valproic acid (VPA), a short-branched fatty acid, has been shown to inhibit chromatin remodeling class I histone deacetylase (HDAC I) which is involved in tumor development. A novel drug, ACS2, coupled by H_2_S releasing fraction and VPA, induces apoptosis by mitochondrial pathway in NSCLC as well as inhibits cancer cell invasion and metastasis by down-regulating matrix metalloproteinase-1 expression and enhances the sensitivity of lung cancer cells to cisplatin [131].

### Nanoemulsions for H_2_S release and probes for H_2_S sensing

BAD-NE, synthesized from BSA/α-linolenic acid (ALA)/diallyl disulfide (DADS), is a nanoemulsion capable of releasing H_2_S, which has been shown to induce cell cycle arrest by promoting p21 expression and apoptosis by activating the ERK1/2 signaling pathway in both MCF-7 breast cancer and HuT 78 T-cell lymphoma cells [132]. A novel H_2_S-sensing bifunctional fluorescent probe for H_2_S-sensing based on naphthalimide peptide coupling, can be activated by endogenous H_2_S in cancer cells and could serve as a diagnostic molecule for high H_2_S-expressing cancer cells and induce apoptosis at the same time [133]. CPC is also a novel H_2_S probe that reacts with endogenous H_2_S to reduce H_2_S concentration, decrease the glutathionylation of Keap1 at Cys434, and increase the interaction between Keap1 and Nrf2, thereby inhibiting nuclear translocation of Nrf2, suppressing autophagy and promoting apoptosis [134].

## Apoptosis induced by natural organic H_2_S donors in cancer cells

**Fig. 4:**
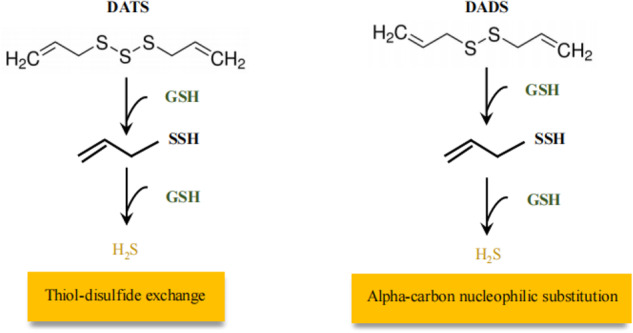
The ways of DATS and DADS in the generation of H_2_S. DATS and DADS are two main active ingredients in garlic. DATS reacts rapidly with GSH to release H_2_S via thiol−disulfide exchange followed by allyl perthiol reduction by GSH. DADS only releases a minute amount of H_2_S via a sluggish reaction with GSH through an α-carbon nucleophilic substitution pathway. DATS diallyl trisulfide, DADS diallyl disulfide, GSH glutathione.

There are many natural organic H_2_S donors in natural world, such as the rocket or broccoli plant family [135]. Natural sulfides are mainly derived from garlic, diallyl sulfide (DAS), DADS, and DATS. It has been reported that garlic-derived organic polysulfides induce H_2_S production in a thiol-dependent manner in erythrocytes [136]. DATS reacts rapidly with GSH and releases H_2_S via thiol-disulfide exchange. DADS releases trace amounts of H_2_S via a slow reaction with GSH via the α-carbon nucleophilic substitution pathway [137–141] (Fig. 4). Garlic derivatives such as DATS and DADS are not only H_2_S donors, but also form a positive feedback loop for H_2_S production by acting with the H_2_S/CBS or H_2_S/CSE axis [142–144]. There are many studies related to the promotion of cancer cells by natural sources of H_2_S. They are summarized in the following Table 2.

**Table 2:** Organic H_2_S donors induce apoptosis in cancer cells through multiple mechanisms.

Cancer types	Mechanisms	References
Pancreatic cancer	DATS: Blocks cell cycle and induces apoptosis	[68]
Esophageal cancer	DADS: Promotes p53 expression and inhibits MEK/ERK pathway via endogenous apoptosis pathway	[179, 180]
Lung cancer	DADS/DATS: Endogenous apoptosis pathway	[181–183]
Breast cancer	DADS: Activation of MAPK/JNK and p38, inhibition of β-catenin DATS: Activation of JNK, AP-1, ASK1-MEK-JNK-Bim via ROS	[184–189]
Leukemia	DADS: Activation of p38/MAPK and inhibition of ERK1/2 via ROS	[190, 191]
Osteosarcoma	DATS: Inhibition of PI3K/Akt pathway via ROS	[192]
Colorectal cancer	DADS: Via mitochondrial and death receptor apoptosis pathways, Cyt C-mediated mitochondrial non-dependent apoptotic pathway DATS: Via mitochondrial apoptosis pathway	[193–197]
Melanoma	DATS: Induces DNA damage, intracellular Ca^2+^, and mitochondrial damage via the ROS-activated p53 pathway	[198–200]
Bladder cancer	DATS: Inhibition of PI3K/Akt pathway and activation of P38/JNK-MAPK pathway	[201–203]
Hepatocellular carcinoma	DADS: Activation of p38/MAPK-mediated intrinsic apoptotic pathway	[204, 205]
Prostate cancer	DATS: Activation of JNK/MAPK pathway induces intrinsic apoptosis pathway, inhibition of Akt activation by the mitochondrial pathway DADS: Ca^2+^-dependent apoptosis, pro-apoptosis via activation of JNK and inhibition of PI3K/AKT signaling	[206–212]
Gastric cancer	DATS: Activation of JNK/p38 MAPK pathway, inhibition of PI3K/Akt-Nrf2 pathway, induction of ROS-mediated AMPK phosphorylation	[213, 214]
Glioblastoma/neuroblastoma	DAS,DADS,DATS: ROS can activate p38/JNK signaling pathway and induce endoplasmic reticulum stress DAS,DADS: Activation of Ca^2+^/calpain/caspase-3 DATS: High expression of ROS and p53 enhance TRAIL-mediated extrinsic apoptotic pathway	[215–219]
Thyroid cancer	Induction of mitochondrial apoptosis pathway by activation of MAPK pathway	[220–222]
Cervical cancer	Induction of mitochondrial apoptotic pathway and cell cycle arrest via p53 pathway	[223]

*ADA* acetyl deacylasadisulfide, *COX-2* cyclooxygenase-2, *Cyt C* cytochrome C, *DAO* d-amino acid oxidase, *DADS* diallyl disulfide, *DAS* diallyl sulfide, *DATS* diallyl trisulfide, *ERK* extracellular signal-regulated kinase, *ERs* endoplasmic reticulum stress, *JNK* C-Jun N-terminal kinase, *KEAP1* Kelch Like ECH Associated Protein 1, *MAPK* mitogen activated protein kinase, *ROS* reactive oxygen species, *PI3K* phosphoinositide 3-kinase, *STAT* signal transducer and activator of transcription 3, *TNF* tumor necrosis factor.

Acetyl deacylasadisulfide (ADA) is also a naturally occurring H_2_S donor. ADA inhibits the PI3K/Akt pathway and its downstream target NF-κB in melanoma cells, reduces the expression of the anti-apoptotic proteins c-FLIP, XIAP and Bcl-2, and promotes the activation of caspase-3 and PARP to induce apoptosis [145]. 12b is a novel synthetic H_2_S donor derived from natural ent-kaurane diterpenoid oridonin derivatives that can induce apoptosis in a variety of cancer cells (e.g. HepG2, HCT-116 and K562 cells) via extrinsic and intrinsic apoptotic pathways [146]. Another experiment demonstrates that in a variety of human cancer cell lines 12b inhibits the cell cycle and induces the mitochondrial apoptotic pathway via the release of H_2_S [147]. Erucin, a diet-derived H_2_S donor, can inhibit the proliferation and metastasis by suppressing calmodulin and the transcription factor expression during epithelial-to-mesenchymal transition (EMT) of melanoma cells [148]. Activating mutation of KRAS in pancreatic cancer cells AsPC-1 leads to hyperphosphorylation of ERK1/2 kinase, causing proliferation and growth of pancreatic cancer cells [149]. Erucic acid can directly induce apoptosis by releasing H_2_S and inhibit cell proliferation by inhibiting the phosphorylation of ERK1/2 in AsPC-1 cells.

## H_2_S as a shield

It has been shown that CSE promotes intra-mitochondrial H_2_S production through translocation to mitochondria that can maintain mitochondrial production of ATP under hypoxic condition [150]. Suitable concentrations of H_2_S in the mammalian colon can serve as a substrate for the mitochondrial oxidative respiratory chain [151]. In particular, 3-MST modulates H_2_S production in mitochondria and is involved in complementing and balancing the bioenergetic role of Krebs cycle-derived electron donors [152]. Some teams have demonstrated that the CSE/H_2_S axis promotes mitochondrial biogenesis in hepatocytes by enhancing the expression and activity of PGC-1α [153]. CBS expression is upregulated in colon cancer cells. CBS produces H_2_S at low to moderate levels in the early stage of cancer development, which acts as a bioenergetic factor for cancer cell growth and promotes vasorelaxation generation, supporting tumor growth and proliferation [12, 90]. There is a positive feedback loop between H_2_S and nicotinamide phosphoribosyl transferase (Nampt) that regulates the dedifferentiation of cancer cells and helps them recover from potentially lethal damage [154]. Bhattacharyya S et al. find that CBS co-localizes with mitochondrial markers in ovarian cancer, and silencing CBS inhibits mitochondrial respiration and ATP synthesis, while increases ROS production. Mechanistically, silencing CBS reduces H_2_S production, severely decreases cellular GSH levels, activates tumor suppressor p53 and inhibits NF-κB [95]. H_2_S ameliorates hypoxia and oxidative stress-induced injury because of its ability to scavenge intracellular ROS and increase GSH level [155–157]. Exogenous application of 100  μM NaHS to pancreatic β cells inhibits cytokine-induced ROS production [158]. H_2_S is a vital endothelium-derived hyperpolarizing factor (EDHF) which activates ATP-sensitive, medium and small conductance potassium channels through cysteine S-sulfhydration, leading to hyperpolarization and vasorelaxation of vascular endothelial and smooth muscle cells [159]. CBS silencing inhibits tumor growth and neointimal density in rat models of colon and ovarian cancer, and low concentrations or endogenous H_2_S can promote tumor growth by promoting angiogenesis [12, 95]. An exogenous slow-releasing H_2_S donor, GYY4137 inhibits TNF-α-mediated endothelial cell death by S-sulfhydration of pro-caspase 3 leading to down-regulation of its activity [160]. Oral squamous cell carcinoma (SCC) is applied with low concentrations of NaHS, p-Akt and p-ERK1/2 expression is up-regulated in the NaHS-treated group compared with untreated controls, and induces more SCC cells to enter the S phase to promote cell proliferation [161]. 50–200 μM NaHS also induces proliferation of human colon cancer cells by inducing Akt and ERK phosphorylation, inhibiting p21 expression and NO production [162].

Some studies have investigated the protective mechanisms of H_2_S in different cancer cells. Jingfu Chen [163] demonstrates that the p38 MAPK/ERK1/2-COX-2 pathway is involved in NaHS-induced proliferation and anti-apoptosis in C6 glioma cells. H_2_S not only inhibits bronchial epithelial cell apoptosis through modulation of ER stress, but also inhibits NOD-like receptor pyrin domain containing protein 2 inflammatory vesicle formation via the Nrf2-dependent pathway [164, 165]. In the HCC cell line PLC/PRF/5, exogenous application of 500 μM NaHS inhibits caspase-3 production and activates the NF-κB pathway to promote cell proliferation [62]. It also inhibits apoptosis by activating STAT3/COX-2 signaling pathway [166]. In esophageal cancer EC109 cells, application of 400 μM NaHS significantly inhibits apoptosis by activating NF-κB and p38 MAPK/ERK1/2-COX-2 signaling pathways [54]. Similarly, 500  μM NaHS acts on multiple myeloma cells for 24 h can promote cell proliferation and migration by upregulating p-Akt expression [167].

## Discussion

**Fig. 5:**
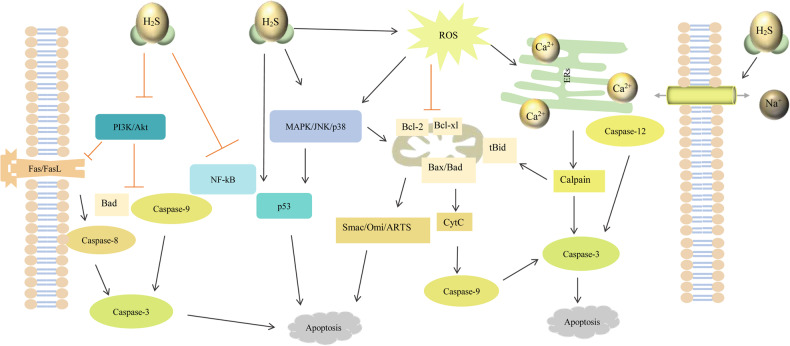
H_2_S regulates three pathways of apoptosis through multiple signaling pathways. Cyt C cytochrome C, Fas fas cell surface death receptor, Fasl fas ligand, Smac second mitochondria-derived activator of caspases, JNK C-Jun N-terminal kinase, MAPK mitogen activated protein kinase, NF-κB nuclear factor-kappa B, PI3K phosphoinositide 3-kinase, Akt protein kinase B, ERs endoplasmic reticulum stress, ROS reactive oxygen species.

In summary, we know that H_2_S is a substance with small molecular mass that can freely penetrate lipid membranes and has the redox property, it exerts an anti-apoptotic effect in cancer cells via a variety of mechanisms (Fig. 5). Firstly, H_2_S, at low concentrations, scavenges ROS via its inherent reducing property and by elevating intracellular levels of GSH through the activation of cysteine/cystine transporter protein. Furthermore, H_2_S can directly activate K^+^_ATP_ channel, induce vascular smooth muscle relaxation and promote local blood supply to tumors. Mustafa et al. report that H_2_S activates glyceraldehyde 3-phosphate dehydrogenase (GAPDH) via S-sulfhydration under hypoxic conditions, which is involved in glucose metabolism, and increases the enzymatic activity of GAPDH to promote anaerobic metabolism [168]. Lower concentrations of H_2_S also stimulate oxidative phosphorylation and increase ATP production by donating electrons to the mitochondrial electron transport chain. In addition to acting as a signaling molecule, H_2_S regulates apoptosis in cancer cells via S-sulfhydration of amino acid residues of certain protein molecules in signaling pathways. For example, ERK, JNK, and p38 [169–172], which control numerous pathophysiological processes, ERK regulates cell growth and differentiation, as well as JNK and p38 play important roles in inflammation and apoptosis. The PI3K/Akt pathway is another important intracellular signaling pathway that is significantly associated with tumorigenesis, cancer progression and drug resistance [173, 174]. It also responds to intra- and extra-cellular signals to promote metabolism, proliferation, cell survival, growth, and angiogenesis [175]. Mutations in the PI3K/Akt pathway are common phenomena in human cancers, and the two most frequently mutated genes are phosphatase and tensin homolog and PI3K-alpha. This pathway is able to inactivate the pro-apoptotic factors Bad and pro-caspase 9 and inhibit the expression of the death ligand FasL [176]. H_2_S directly sulfurizes the upstream and downstream of the p53 signaling pathway, NF-κB, and Keap1 to inhibit cell proliferation and induce apoptosis. H_2_S not only directly regulates various intracellular signaling pathways but also participates in the regulation of tumor microenvironment [177, 178]. Therefore, there is an urgent need for more effective methods to detect the concentration of H_2_S in cancer cells. The H_2_S level is then used as a guide for the development of novel H_2_S-releasing drugs, as well as the inhibitors/activators of H_2_S-producing enzymes. In conclusion, H_2_S can be applied in cancer treatment by identifing the mechanism of H_2_S in the apoptotic process of cancer cells.
